# Emergency response in overturned pig transport vehicles: Description and discussion of Danish cases from a One Welfare perspective

**DOI:** 10.1017/awf.2025.6

**Published:** 2025-02-10

**Authors:** Cecilie Kobek-Kjeldager, Kirstin Dahl-Pedersen, Mette S Herskin

**Affiliations:** 1 Department of Animal and Veterinary Sciences, Aarhus University, DK-8830, Tjele, Denmark; 2 Department of Veterinary Clinical Sciences, University of Copenhagen, DK-2630 Taastrup, Denmark

**Keywords:** animal welfare, emergency management, in-transit, livestock, road accident, traffic accident

## Abstract

Based on experiences from stakeholders, this paper describes and discusses Danish emergency procedures when animal transport vehicles overturn, from a One Welfare perspective. Twenty qualitative interviews were conducted with selected stakeholders involved in emergency responses and their co-ordination. Results from interviews were extracted and are presented as a description of the Danish emergency management procedures in situations where pig transport vehicles overturn in a traffic accident. The description is followed by a discussion of six identified themes related to animal welfare and One Welfare in such situations: (1) Standard operating procedures; (2) Balancing animal welfare and work safety; (3) Roles, education and experience; (4) Communication, time and access to animals; (5) Debriefing; and (6) Killing of animals. Overall, the analyses of the interviews showed that the emergency response at an overturned pig transport vehicle involves different professional groups, requires technical knowledge regarding animal transport vehicles as well as knowledge of the species involved and how to handle the animals. The results are discussed from a One Welfare perspective, suggesting that these emergency responses include an inherent societal prioritisation dilemma involving the balancing of, for example, training, preparation and debriefing of different professional groups. Further research is needed to address ethical considerations, share best practices, and enhance emergency protocols.

## Introduction

During the period from 2017 to 2021, approximately 2.5 billion live farmed animals were transported annually between EU member states (European Court of Auditors 2023a). In the same period, Denmark exported 76–89 million farmed animals per year (including approximately 78 million poultry, 15 million pigs, and 59 thousand cattle). The majority of these consignments involved road transportation (European Court of Auditors 2023b), which means that a significant number of vehicles carried farmed animals on Danish and European roads. While in traffic, any vehicle is at risk of being involved in an accident (Prato *et al.* 2014). When an animal transport vehicle overturns in a traffic accident, the consequences may be severe, for the animals, the livestock driver, other people directly involved as well as the emergency personnel. Additionally, like other traffic accidents, crashed animal transport vehicles can negatively impact other road users and traffic as such (Másilková 2017). In situations like this, access to a quick and efficient emergency response is critical (Rembalovich *et al.* 2020).

To the best of our knowledge, very few scientific papers have focused on accidents involving vehicles transporting farmed animals (Woods & Grandin 2008; Miranda-de la Lama *et al.* 2011), and they mainly discussed the prevalence of such accidents and not the consequences of, or access to, emergency responses. Miranda-de la Lama *et al.* (2011) reported the prevalence of accidents in Spain from 2000–2008 (mainly involving pigs), while Woods and Grandin (2008) reported accidents in the US and Canada from 1994–2007 (mainly involving cattle). In both papers, transport vehicles overturning was found as the major accident type. In a questionnaire survey on occupational hazards among livestock drivers transporting sheep in Mexico, Valadez-Noriega *et al.* (2018) mentioned traffic accidents as the second largest reported risk (reported by 43%). More recently, Anneberg and McLoughlin (2022) performed a semantic analysis, discussing the choice of words in European media, when reporting from accidents involving overturned animal transport vehicles. The authors were critical of the language used by the media in these situations, since the media portrayals were interpreted as reinforcing a commodified view of farm animals.

During recent years, animal disaster management in general has been subject to increased attention from scientists (e.g. Anthony & De Paula Vieira 2022), from international organisations such as WOAH (https://www.woah.org/en/what-we-offer/emergency-preparedness/), in media targeting farmers or agribusiness (e.g. Farmprogress 2021) or as courses offered in animal rescue training for private persons (ASAR 2024). This focus seems, however, to be directed most often at either one-animal accidents (such as injured horses) or at larger scale disasters, such as disease outbreaks, war or natural disasters. It has not been possible to find scientific literature discussing the emergency management when a traffic accident involving an animal transport vehicle has taken place. Protocols for emergency management in such cases can be found in two German non-scientific publications (Gayer *et al.* 2016; Mitglieder der Länderarbeitsgruppe 2022) and in a support document originating from the Swedish University of Agricultural Sciences (Anonymous 2016), but those documents do not contain discussion of the management as such.

In terms of the accidents involving transport of farmed animals, the incidents will have negative consequences both for the welfare of the animals involved and as well as possibly affecting humans. The risks of these consequences can be interconnected. According to the One Welfare principle (i.e. the interconnections between animal welfare, human well-being and the environment), examination of issues related to the welfare of humans as well as non-human animals often require a broader interdisciplinary approach (Colonius & Earley 2013; Pinillos *et al.* 2016; Bourque 2017), as these areas share scientific measurement methods, overlap and influence each other. For this reason, the aim here was to incorporate aspects of both the welfare of farmed animals and human well-being, describing and discussing the emergency response in cases of overturned animal transport vehicles from a One Welfare perspective. The available material, based on a number of Danish cases, was collected as qualitative interviews with selected stakeholders.

## Materials and methods

In Denmark, since 2017, the emergency efforts when pig transport vehicles are involved in traffic accidents where the vehicle overturns have been co-ordinated by an ‘emergency management group’, overseen by the Danish pig industry association. Members of the group include representatives from the police, the Fire & Rescue Service, the Danish Veterinarian Association, the Danish pig transport industry, the Danish Road Directorate, the Danish Road Traffic authorities and the Danish Veterinary and Food administration.

This study involved 20 qualitative interviews with 19 selected Danish stakeholders and one German stakeholder, representing central actors in the emergency response.

The Danish stakeholders were persons directly involved in emergency responses and/or their co-ordination, including: (a) three representatives from the police; (b) four from the Fire & Rescue Service; (c) four veterinarians, including a representative from the Danish Veterinarian Association; (d) a representative from a large Danish pig industry association (Danish Agriculture and Food Council); as well as (e) two representatives from the Danish pig transport industry; and (f) five livestock drivers. The livestock drivers had themselves been in an accident or been summoned to assist with a replacement vehicle. In addition, an interview was conducted with a representative from the German veterinary authorities. This specific interview was included because of the large trade of live pigs from Denmark to other EU Member States (European Court of Auditors 2023a). This trade means that livestock drivers enter or pass through Germany when transporting Danish pigs (Dahl-Pedersen *et al.* 2022). For this reason, it was considered relevant to add information and experiences from Germany. All interviewee identities are known but are not disclosed in this paper.

Initially, interviews were conducted with two participants from the group, one person from the police and one from the Danish pig industry association. These individuals functioned as interview gatekeepers (Andoh-Arthur 2019) for the subsequent snowball sampling process (Naderifar *et al.* 2017). During the interview period, we tried to make effective use of the limited research resources by choosing respondents via so-called purposive sampling (Campbell *et al.* 2020), focusing on actors who had experience of being responsible for decisions related to animals at accident scenes. In total, 14 persons were interviewed regarding their experiences and role at the emergency scenes, and all were initially contacted via email. In addition to the persons involved at the emergency scenes, the interviews involved five livestock drivers who had been involved in accidents themselves or been involved in the emergency response at other accidents. The livestock drivers were contacted and interviewed by telephone as agreed with their employer.

Since the interviewees had quite different roles and thus experiences, at the emergency scenes, they were interviewed in a semi-structured way, with questions focusing on their experiences relating to emergency situations involving pig transport vehicles. Questions consisted of a mixture of quantitative and qualitative and no formal interview guide was used. We were also interested in One Welfare perspectives and potential improvements. We therefore also asked about potential issues and disturbances, for example, from bystanders/journalists taking photographs and in the work with the other professional groups and the interviewees’ suggestions as to how to alleviate it. The use of semi-structured qualitative interviews allowed each interviewee to contribute a new perspective to the overall picture, and interviewees were therefore encouraged to use examples from their experiences and direct the focus of the interview as they so desired within the constraints set (Jowsey *et al.* 2021).

All interviews were carried out by CKK (female, Danish, post doc, focusing on animal welfare during critical life events) from March to September 2023 and could involve physical presence, online meetings or phone. Interviews conducted online or in person were audio-recorded. Phone interviews were summarised in note form and not transcribed *verbatim.* All interviews were transcribed manually to notes that were organised into common themes (Brinkmann & Kvale 2015). During the analysis of the interviews, the emergency response was summarised, and the six themes were identified and extracted.

### Ethical considerations

Although no ethical approval was needed under Danish legislation, we acknowledged the risk of participants to experience psychological effects due to the potentially emotive nature of the topic. To minimise the risk of psychological harm, we implemented the following measures: (1) Voluntary Participation: Participants were initially approached via email or, in the case of drivers, through their workplace supervisors, to assess their willingness to participate; (2) Informed Consent: All participants received information regarding the study’s purpose and methodology, enabling them to make an informed decision about their involvement; (3) Right to Withdraw: Participants had full autonomy to withdraw their consent at any point during or after the interview, without any repercussions; and (4) Privacy Protection: All responses were anonymised to protect participant identities. While certain individuals may be identifiable within their professional community, their anonymity is preserved for the general readership. These individuals consented to this premise.

## Results

**Figure 1.**
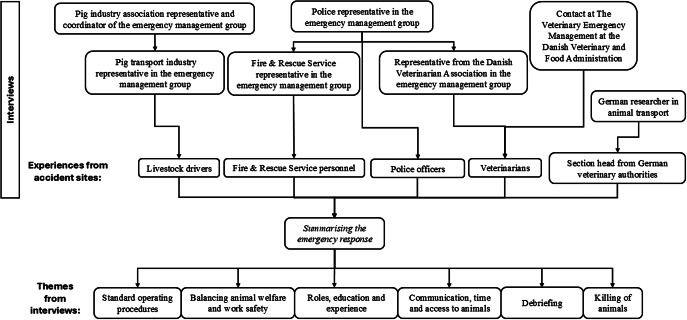
Overview of the interviewed stakeholders and the themes extracted from the summarised emergency response when a pig transport vehicle overturns in Denmark.

Below, we summarise the emergency response in Denmark followed by a discussion of the six themes retrieved from the interviewed stakeholders (Figure 1). The first theme was ‘Standard operating procedures’ which highlights that the co-ordination of the emergency response differs between countries. Significant aspects of human well-being were thematised during the emergency response task in ‘Balancing animal welfare and work safety’ and after the task in ‘Debriefing’ while the competences of the professional groups were thematised in ‘Roles, education and experience’. The theme ‘Communication, time and access to animals’ highlights the major challenges in the emergency response task, while the final theme was the procedure regarding ‘Killing of animals’.

### Overview of the Danish emergency response system

The following description was put together based on input from a total of 20 interviews with different professional groups: livestock drivers involved in accidents themselves or involved in the emergency response at other accidents, professionals involved in the Danish emergency management group and/or with experience from accidents involving pig transport vehicles.

In general, the procedure followed in Denmark in cases of traffic emergencies is for the authorities to be alarted via telephone as soon as the accident has happened or been noticed. The police emergency call centres then adhere to certain protocols in terms of whom to summon to the site of the accident – typically involving police, paramedics and rescue personnel.

Once the emergency management group has been established, the police emergency call centres have a so-called ‘action card’ regarding whom to summon to the site of the accident in cases of an overturned pig transport vehicle. This action card includes summoning Fire & Rescue personnel and contact information for: (1) veterinarians from private practices stratified by geographical region; (2) pig transport companies to summon a replacement vehicle and replacement livestock driver; (3) contact with a rendering company for disposal of dead animals; and (4) the possibility of summoning slaughterhouse personnel for emergency killing. The emergency management group has created a contact list of veterinarians operating on a voluntary basis as well as facilitating access to slaughterhouse staff. The emergency management group has also created an action card specifically for the veterinarian involved. According to this action card, the task of the veterinarian, when present at the scene of the accident, is to assume responsibility for the animals in question and evaluate their health and fitness for further transport. The veterinarian may also assist with the task of emergency killing, but this is not presented as the primary task of this professional group.

According to the procedures developed by the emergency management group, it is the overall responsibility of the police to ensure the accident site is designated an appropriate outer perimeter and traffic is suitably redirected. Meanwhile a task leader of the Fire & Rescue Service has the technical responsibility for an inner perimeter of the accident site. Within the inner perimeter, initial work involves the creation of an overview, securing working conditions, and establishing access to the animals. It may be necessary to brace the vehicle’s floors/ceiling prior to personnel being able to enter. The Fire & Rescue personnel are considered responsible for the rescue of the animals. Manual extraction with ropes or by hand may be necessary. Entry into the vehicle in question may be necessary to enable animals to exit the vehicle. At the site of the accident, the police may summon further products, for example, from nearby building supply stores, to be used as fencing material for animals that have been rescued from the vehicle. The police may use a drone or thermographic camera to search for escaped animals.

The livestock driver involved in the accident will often be brought to an emergency room or hospital due to injuries and/or for a check-up. Although there is much variation between accidents, the livestock driver of the crashed vehicle is, according to our investigation, often not involved in the emergency response as such. Instead, a driver summoned with a replacement vehicle will provide technical assistance regarding the overturned vehicle (e.g. regarding the movable decks present inside most pig transport vehicles) and practical experience on how to move animals into the replacement vehicle.

When the involved animals have been assessed as being fit for further transport by a veterinarian and been moved to the replacement vehicle, the transport company has coordinated the further destination of the animals. If the original destination was a slaughterhouse, the pigs will be sent to the nearest slaughterhouse. Pigs transported for further production (e.g. weaner pigs) can be sent to the nearest assembly barn. Within normal office hours, an official veterinarian will typically be present at the assembly barn and can re-evaluate the animals upon arrival. Animals evaluated as being unfit for further transport will be killed at the assembly barn.

Carcases are retrieved and disposed of by a summoned rendering company. After completing the task, the Fire & Rescue personnel perform a technical debriefing at the site of the accident.

## Discussion

Overall, the qualitative interviews showed that the emergency response in action at an overturned pig transport vehicle involves different professional groups, requires technical knowledge about animal livestock vehicles and how they are operated, as well as knowledge about the animal species in question and how best to handle them. In addition, in terms of emergency rescue operations, overturned pig transport vehicles represent something of an anomaly. Below, we discuss our findings based on the six themes extracted from the interviews in terms of animal welfare as well as the well-being of the professionals involved in the rescue operations (see themes in Figure 1).

### Standard operating procedures

The first theme identified and extracted from the information obtained from the interviews was related to ‘Standard operating procedures’ (Figure 1). In contrast to certain EU Member States, such as Germany, the Danish emergency management when animal transport vehicles overturn, does not fall under the official veterinary emergency response from the Danish Veterinary Authorities (Danish Food and Veterinary Administration). Thus, in such cases, there are no official standard operating procedures available from the competent authorities on animal health and welfare. Instead, Denmark has an industry co-ordinated emergency management group consisting of several stakeholders (see above), established with the overall aim of preventing accidents with vehicles transporting pigs, as well as improving the emergency management when an accident has occurred.

To the best of our knowledge, there are currently no standard operating procedures or ‘best practice’ descriptions of emergency response in cases of overturned vehicles transporting pigs, including emergency killing, to best ensure the welfare of the animals in question. However, European protocols for emergency management in cases of traffic accidents with animal transport vehicles can be found in two German non-scientific publications (Gayer *et al.* 2016; Mitglieder der Länderarbeitsgruppe 2022) and in a support document from the Swedish University of Agricultural Sciences (Anonymous 2016). In North America there seems to be a greater focus on protocols and training from universities and the private sector (Pederson *et al.* 2018; Farmprogress 2021; Anonymous 2022; Farm & Food Care Ontario 2022; Ferry 2024).

Development of standard operating procedures across countries will be complex. Accident sites differ as do access to personnel and equipment (e.g. rural road vs highway, animal number and weight class, vehicle design). Furthermore, the ethical approach to production animals varies both between and within countries, which will have consequences for how animal welfare is prioritised.

Anthony and De Paula Vieira (2022) discussed animal disaster management and suggested that guidance should be developed through an ethics of care approach. The authors argued that the use of this line of ethics – where concern for the welfare of others and nurturing is emphasised as the basis of relationships – has the strength of mobilising relational solidarity during a time of human-animal shared vulnerability to crisis. An accident involving an animal transport vehicle is such a case where human rescue intervention is needed. Based on texts such as Beever and Morar (2019), Anthony and De Paula Vieira (2022) argued – from a One Health perspective – that harms to animals (e.g. disease, injury and death) are strongly correlated with harms to humans.

Currently, to our knowledge, no ethical or animal welfare analyses for animals involved in such accidents in Denmark or internationally are available. Anthony and De Paula Vieira (2022) encouraged the development of best-practice models and inclusive strategies for serving humans, animals and the environment during emergency situations. Further research is needed to clarify whether protocols or other initiatives can be taken to facilitate the quickest and smoothest responses possible – even for personnel without a lot of practical experience – and upon which ethical considerations such decision-making tools should be based.

Based on the current work, the standard operating procedures should include many considerations including prior training of personnel, local communication network (between police, Fire & Rescue Service, veterinarians, animal transport companies, slaughterhouses, and rendering companies), equipment for hauling, confining and emergency killing, work safety, management of traffic flow and bystanders, debriefing and/or psychological assistance. Consideration should also be made regarding the extent to which the same procedures could be applied in instances of vehicles transporting other species (e.g. cattle, poultry, horses, fish) and overturning.

### One Welfare-related themes

#### Balancing animal welfare and work safety

The One Welfare principle highlights the interconnections between animal welfare and human well-being. Such interconnections are clearly present in the emergency response when a pig transport vehicle overturns. It is self-evident that an overturned pig transport vehicle is associated with significant negative welfare for the animals in question. It is therefore crucial to have an emergency preparedness that can handle the situation quickly and efficiently, thereby limiting the negative consequences as much as possible. In the present paper, the One Welfare principle is related to the theme identified as ‘Balancing animal welfare and work safety’. It was clear from the interviews with Fire & Rescue personnel that work safety formed an implicit cornerstone of their approach, while the potential danger of entering an overturned vehicle with, for example, heavyweight slaughter pigs was raised in other professional groups.

Furthermore, the information obtained from the interviews clearly showed the consequences for the involved humans to be categorised as either having a physical or a mental impact. Ideally, the working conditions of a well-functioning emergency response should aim to minimise both (Burke & Richardsen 2019).

The physical strain takes place during the emergency response, but may have longer term consequences, and includes injuries to the livestock driver of the crashed vehicle, as well as the working conditions of the summoned personnel. Interviewed Fire & Rescue Service employees summoned to accident sites described being required to adopt awkward postures with heavy lifting and strenuous dragging to rescue the pigs. Similar aspects are important within the construct of physical workload (da Costa & Vieira 2010; Gentzler & Stader 2010). The physical working conditions for livestock drivers are ergonomically challenging even under normal conditions (Wilhelmsson *et al.* 2019, 2021, 2022, 2023). Further studies are needed to clarify needs for training and equipment as well as replacement personnel, in order to limit the physical strain as much as possible.

The mental health challenges are relevant during, not to mention in the aftermath of, the emergency response and includes the often traumatic experiences of the livestock driver of the overturned vehicle – even in cases of unconsciousness and memory loss from the exact time of the accident (Mayou *et al.* 2000). In a questionnaire survey of international livestock drivers, the respondents highlighted the experience of excessive responsibility, for example, in regard to the evaluation of animal fitness for transport, causing stress and anxiety over potential mistakes (Dahl-Pedersen *et al.* 2022). From general descriptions, not involving livestock vehicles, typical psychological consequences of being in an accident are known to be shame and guilt. How to process this has relevance for subsequent mental well-being (Ho *et al.* 2000). According to Ho *et al.* (2000), accepting responsibility for a negative outcome can constitute a kind of coping mechanism aimed at re-establishing control and boosting one’s resistance to stressful situations. Currently, there are no studies available on the mental health challenges experienced by livestock drivers after being involved in accidents, and thus no tools are available to facilitate the recovery process in the post-accident period.

The mental health challenges associated with such accidents extend to the professionals summoned for the emergency response as well as potential bystanders. Among evident mental burdens, which were more or less articulated in the interviews and are also known from the scientific literature concerning farmed animals, are time pressure (see also Gerritzen & Raj 2009), waiting time (e.g. if a recovery vehicle is needed), the feeling of a lack of power when witnessing/sensing animals suffering, organisational issues, and/or lack of resources (Jacobsson *et al.* 2015; Burke & Richardsen 2019). The complexity of rescue tasks, especially in cases such as rescues after accidents involving vehicles transporting animals, may be among the reasons why firefighters have reported assessing ‘accidents’ as more stressful than ‘fires’ (Rodrigues *et al.* 2018).

Further categorised under the theme ‘Balancing animal welfare and work safety’, the interviewees present at accident sites were asked about whether their work had been disturbed by bystanders/journalists taking photographs. While such presences had been noticed by some, but not all, it was not considered an issue. In the case of bystanders, they adhered to the instructions from Fire & Rescue personnel to keep a respectful distance.

#### Roles, education and experience

Overall, the infrequency of accidents involving animal transport vehicles in terms of emergency responses, means that professionals will not encounter such events too often during their career and, as such, the required responses are unlikely to ever become routine (see also Gavinelli *et al.* 2014). In cases like this, Anthony and De Paula Vieira (2022) described how an emergency management cycle should be structured to include four key phases: planning, preparedness, response and recovery. In the current study, a major challenge raised by the interviewees was how to be efficiently prepared for rare events like an overturned pig transport vehicle which most task leaders, veterinarians and livestock drivers may never encounter. This challenge can be included in the theme ‘Roles, education and experience’, and poses an inherent societal prioritisation dilemma involving the balancing of training and preparation for other types of emergencies that occur at a higher frequency, or which may involve animals with higher economic, emotional, and/or ethical value in society at large, such as accidents with horse trailers. According to Danish information available in Kobek-Kjeldager *et al.* (2024), during the last seven years, 2–9 accidents involving pig transport vehicles have taken place each year in Denmark, involving different categories of pigs transported for slaughter or for further fattening. The accidents typically involved a relatively high number of animals each (median: 220, min–max: 35–700) and was associated with a considerable mortality (median: 30%, min–max: 0–77% pigs either died during the accident or were killed on-site as they were not considered fit for further transport). Though the total number of animals involved in accidents with animal transport vehicles in Denmark is small compared to, for example, the number of animals involved in natural disasters such as wild fires in other geographical regions (Cowled *et al.* 2022), animal welfare is seen as an individual attribute (Arndt *et al.* 2022). Thus, it may be considered crucial, both ethically and for research, to consider not simply the number of individuals but also the welfare level of individual animals.

It may pose challenges when professional groups with distinct skillsets, and who rarely under normal circumstances work alongside each other, are compelled to work together under pressure such as during emergency responses. Some of the interviewed task leaders from the Fire & Rescue Service called for more educational focus as well as more training on animal rescue, where practical training (as described in ASAR 2024 regarding accidents with large farm animals) was highlighted as particularly beneficial. A number of the interviewees who were working with animals on a daily basis highlighted a lack of animal handling expertise by Fire & Rescue personnel. Thus, this training may be focused on decision-making and/or animal handling skills, depending on the educational level of the trainees. Across the available literature on animal emergencies, this focus on training is mentioned repeatedly (Gerritzen & Raj 2009; EFSA AHAW Panel 2020; Anthony & De Paula Vieira 2022). More research is needed to investigate the most effective use of resources in this respect.

#### Communication, time and access to animals

Within the theme, ‘Communication, time and access to animals’ errors in communication were described as a cause of increased delay in accessing animals at two accident sites. While the need for clear communication and co-ordination was underlined, all professional groups at the accident sites described a respectful and constructive collaboration. According to the experiences of interviewees at accident sites, the most time-consuming task entailed gaining access to the animals in the vehicle, a process that could take upwards of 1.5 h from the time of the accident until the first animal was rescued. Several interviewees were dissatisfied with the duration of time passing and called for ways to reduce it. Often accidents involving livestock vehicles take place in relatively localised geographical areas – in this case Central Jutland in Denmark – and it may be beneficial to prioritise training of staff and access to materials in such regions. From both Danish data and the available Spanish study (Miranda-de la Lama *et al.* 2011), it appears that accidents with vehicles carrying animals tend to be seen in regions of countries where animal density is high and/or in regions that function as passageways to national borders. In terms of equipment, those accidents where it was deemed necessary to summon a recovery vehicle to restore the overturned vehicle, were described as particularly time consuming. Therefore, an evaluation of the regional localisation and availability of such vehicles may be an opportunity to reduce the duration of the rescue operation. One aspect of the emergency responses that should also be trained for, and where there is a risk of trauma for involved personnel (Slade & Alleyne 2023), is the killing of animals which constituted another theme extracted from the interviews (discussed below).

#### Debriefing

A significant point highlighted in the interviews was varying opportunity to partake in debriefings for the different professional groups. The involved livestock drivers in particular stood out as lacking such opportunities. Debriefing refers specifically to a process whereby an evaluation and discussion of an event or activity takes place, generally following its completion (Kikkawa & Mavin 2017). The purpose typically being to gather feedback, reflect on experiences, and identify future areas for improvement (Fullerton *et al.* 2000; van Emmerik *et al.* 2002; Rose *et al.* 2003). In the interviews forming the basis of this paper, all professional groups were asked about their participation in debriefing activities after being involved in an accident with an animal transport vehicle, as well as the extent to which such debriefings were formalised. The staff from the police and Fire & Rescue Service reported having access to formalised technical debriefings and, in addition, police staff noted having been offered regular psychological supervision. The two remaining professional groups, the summoned veterinarians and livestock drivers, described options for debriefing best characterised as so-called ‘natural debriefings’, involving interpersonal processes with colleagues, friends, and partners when discussing a lived experience (Fullerton *et al.* 2000). The livestock drivers included in the study who had been involved in accidents or assisted in post-accident efforts, generally described their options for debriefing as being somewhat less structured than those of other professional groups. This was especially pronounced for the livestock drivers who had been operating the vehicle in question, which may be somewhat surprising. However, the extent to which this finding can be generalised is not known.

The involved professional groups differed clearly in their options for post-event debriefing but also fulfilled very different roles during emergency responses.

#### Killing of animals

During the interviews, it came to light that although the summoned veterinarian was afforded the task of evaluating the clinical condition of the animals in question as well as their potential fitness regarding further transport, it was not their responsibility to oversee any killing as such. Further, it was also revealed that prior to the arrival of the veterinarian, it could be necessary for the first responders (police and/or Fire & Rescue Service personnel) to make an initial evaluation and initiate emergency killing using firearms. In the EU, ‘emergency killing’ is defined as the killing of animals who are injured or have a disease associated with severe pain or suffering and where there is no other practical possibility to alleviate this pain or suffering (Council Regulation [EC] No 1099/2009). In the German implementation instruction (Mitglieder der Länderarbeitsgruppe 2022), the necessity of emergency killing using police firearms or by hunters is described, while emergency killing by a qualified individual is preferred, specifically a veterinarian. The methods described in the instruction are: (A) ruminants or pigs: stunning with a captive-bolt gun and subsequent killing with/without exsanguination; and (B) pigs: using electric tongs. The Swedish support document (Anonymous 2016) described similar methods for emergency killing for different species but also included lethal injections administered by a veterinarian. As described by the American Veterinary Medical Association [AVMA] (2020) and also by Gerritzen and Raj (2009), the majority of these methods require direct contact with the animal and a certain degree of restraint, which may present a challenge under emergency conditions. In addition, adult pigs have been described by the Humane Slaughter Association as a difficult animal species to shoot with a penetrative captive-bolt gun (HSA 2017) due to the anatomical features of the forehead and brain. The European Food Standards Authority (EFSA AHAW Panel 2020) described the use of firearms as quick and effective and possible to implementfrom a distance. It is, however, also mentioned that the use of firearms in enclosed spaces, or when animals are on hard surfaces, could result in ricochet of free bullets and needs to be done with extreme care in order to avoid injury to humans or any other animals in the vicinity. Irrespective of the method of killing, inexperienced personnel are mentioned as important risk factors during the killing of animals (Gavinelli *et al.* 2014).

In this paper and the related interviews, we focused on the killing method and not so much the decision of whether or not to kill. A comprehensive analysis of the decision to kill requires a deeper exploration of ethical, economic, and legal aspects which lies outside the scope of this paper. In a historical review of the veterinary profession, Otten (2023) noted a shift in killing responsibility over time from owners to become societal obligations for specific professional groups like veterinarians and farmers. According to one of the interviewed assisting veterinarians in our study, who had been working at several accidents, some veterinarians may be hesitant to join on-call lists for larger killing tasks in emergencies. Further research is needed to investigate this potential challenge to emergency responses.

It is noteworthy that in the EU regulation (Council Regulation [EC] No 1099/2009), the chapter on emergency killing states, “*In the case of emergency killing, the keeper of the animals concerned shall take all the necessary measures to kill the animal as soon as possible*”. Neither the Danish regulation (BEK nr 817 af 15/06/2023) nor a recent EFSA opinion on the killing of pigs (EFSA AHAW Panel 2020) mentions emergency killing in the context of accidents involving animal transport vehicles. It is well established that the killing of animals outside of slaughterhouses is associated with significant logistical, personnel, and equipment demands to ensure proper implementation – for both animals and surrounding people (Gerritzen & Raj 2009; Gavinelli *et al.* 2014). In their review of animal killing during disease outbreaks, Gerritzen and Raj (2009) were unable to identify agreed physiological indicators of death with the authors calling for the development of protocols on how to confirm death of animals during emergency killing. Somewhat comparably, Gavinelli *et al.* (2014) requested the development of systems to evaluate the welfare of animals involved in mass killings and suggested that tools and check lists should be provided by competent authorities. In connection with the killing of animals for reasons other than slaughter, the EFSA (AHAW Panel *et al.* 2020) recommended the establishment of standard operating procedures, and that these procedures should be based on the use of animal-based measures (ABMs).

Recently, Ursinus *et al.* (2024) drew attention to a relatively neglected area of animal welfare: the study of the death of animals occurring outside the normal context of a slaughterhouse. The present study has shed light upon another context for this, one where both information and access to data are also limited: when animal transport vehicles overturn in traffic accidents. In order to create an overview of these types of events that may have severe consequences for animal welfare, as well as facilitate efficient knowledge-exchange and cross-country implementation, these experiences gathered from our Danish cases illustrate the potential benefits from having access to EU-wide information on traffic accidents involving animal transport vehicles. Ideally this would be for all commercially transported species and not just pigs, not to mention standard operating procedures that include emergency killing in such situations.

## Animal welfare implications and conclusion

An overturned pig transport vehicle leads inherently to significant welfare issues for the animals involved. Thus, a swift and efficient emergency response is crucial to minimise the resulting negative welfare. This paper has been the first to describe the emergency procedures when animal transport vehicles overturn from a One Welfare perspective – based on a number of Danish cases. The available material was collected as qualitative interviews with selected stakeholders involved in emergency responses and their co-ordination and included 20 interviewees. Overall, the analyses of the interviews show that the emergency response required at an overturned pig transport vehicle involves different professional groups, requires technical knowledge regarding animal livestock vehicles and how they are operated, as well as knowledge about the animal species involved and how to handle the animals. These emergency responses include an inherent societal prioritisation dilemma involving the balancing of training, preparation and debriefing of different professional groups with the negative welfare experienced by the trapped animals. Further research is required, including ethical considerations, as well as sharing of ‘best practice’ and available data, to clarify whether protocol development or other initiatives can be taken to facilitate the quickest and smoothest responses possible.
